# Automated quantification of penile curvature using artificial intelligence

**DOI:** 10.3389/frai.2022.954497

**Published:** 2022-08-30

**Authors:** Tariq O. Abbas, Mohamed AbdelMoniem, Muhammad E. H. Chowdhury

**Affiliations:** ^1^Weill Cornell Medicine-Qatar, Ar-Rayyan, Qatar; ^2^Urology Division, Surgery Department, Sidra Medicine, Doha, Qatar; ^3^College of Medicine, Qatar University, Doha, Qatar; ^4^Department of Electrical Engineering, Qatar University, Doha, Qatar

**Keywords:** penile curvature, artificial intelligence, machine learning, hypospadias, chordee

## Abstract

**Objective:**

To develop and validate an artificial intelligence (AI)-based algorithm for capturing automated measurements of Penile curvature (PC) based on 2-dimensional images.

**Materials and methods:**

Nine 3D-printed penile models with differing curvature angles (ranging from 18 to 88°) were used to compile a 900-image dataset featuring multiple camera positions, inclination angles, and background/lighting conditions. The proposed framework of PC angle estimation consisted of three stages: automatic penile area localization, shaft segmentation, and curvature angle estimation. The penile model images were captured using a smartphone camera and used to train and test a Yolov5 model that automatically cropped the penile area from each image. Next, an Unet-based segmentation model was trained, validated, and tested to segment the penile shaft, before a custom Hough-Transform-based angle estimation technique was used to evaluate degree of PC.

**Results:**

The proposed framework displayed robust performance in cropping the penile area [mean average precision (mAP) 99.4%] and segmenting the shaft [Dice Similarity Coefficient (DSC) 98.4%]. Curvature angle estimation technique generally demonstrated excellent performance, with a mean absolute error (MAE) of just 8.5 when compared with ground truth curvature angles.

**Conclusions:**

Considering current intra- and inter-surgeon variability of PC assessments, the framework reported here could significantly improve precision of PC measurements by surgeons and hypospadiology researchers.

## Introduction

Penile curvature (PC) denotes an abnormal bending of the penile shaft that can occur in either congenital or acquired pathologies of the male external genitalia. The most common underlying congenital pathology is hypospadias which occurs in ~1:250 male live births, with roughly one quarter to one third of cases also displaying substantial PC (Baskin et al., 1996; Stojanovic et al., 2011; Abbas and McCarthy, 2016). Hypospadias-associated PC is caused by arrest in embryological development of the ventral axis of the penile shaft, with features including penile skin deficiency, aberrantly short urethral plate, and ventro-dorsal corporeal disproportion (Keays and Dave, 2017). Degree of PC can significantly influence hypospadias severity (Merriman et al., 2013; Abbas et al., 2020b), and consequently impacts on surgical decision making and ultimate choice of repair procedure (Pippi Salle et al., 2016; Snodgrass and Bush, 2017). If not appropriately managed, PC persists into adulthood and is associated with significant patient dissatisfaction and sexual difficulties (Schlomer et al., 2014; Abbas et al., 2020a; Andersson et al., 2020).

Although PC has significant prognostic value (Merriman et al., 2013), and clinical impact (Spinoit et al., 2020), appraisal of this condition is nor performed consistently between surgeons, and no reproducible tools are available for the rapid assessment of PC extent (Villanueva, 2019). Historically, PC is measured during artificial erection using a saline injection method introduced by Gittes and McLaughlin in 1974 (Gittes and McLaughlin, 1974). However, recent progress in artificial intelligence (AI) and deep learning approaches have enabled automatic segmentation, classification, registration, and analysis of medical images (Litjens et al., 2017). AI has already been applied to multiple different subspecialties in urology, offering high-precision results that could lead to diagnostic and therapeutic benefits (including in endourology, reproductive medicine, stones, hydronephrosis, malignancies, and pediatric urology) (Eun et al., 2021; Hameed et al., 2021). AI displays superior accuracy to traditional statistical methods and could potentially revolutionize clinical decision-making by urologists, especially once these approaches are incorporated into the relevant guidelines (Shah et al., 2020; Catto et al.).

Current evaluation methods for PC include unaided visual inspection, goniometer, and mobile application-based manual angle measurements. However, all of these techniques inherently suffer from high subjectivity and low inter-and intra-observer agreement (Kelâmi, 1983; Villanueva, 2019; Mosa et al., 2022). Penile curvature measurement takes place during the surgery under artificial erection simulation where normal saline is being injected into the corporeal bodies of the penis. Therefore, this is a time sensitive task where getting the correct measurement of PC needs to be done in real-time to reduce the operative time and limit leakage of the normal saline fluid from the surgical field. In the current report, we introduce a novel technology for automatic quantification of PC degree (AccuCurve). Unlike previous studies of PC angle extent measurement, we utilized a segmentation neural network to facilitate rapid angle calculation, potentially leading to a significant time saving for the urologic surgeons.

## Methods

**Figure 1:**
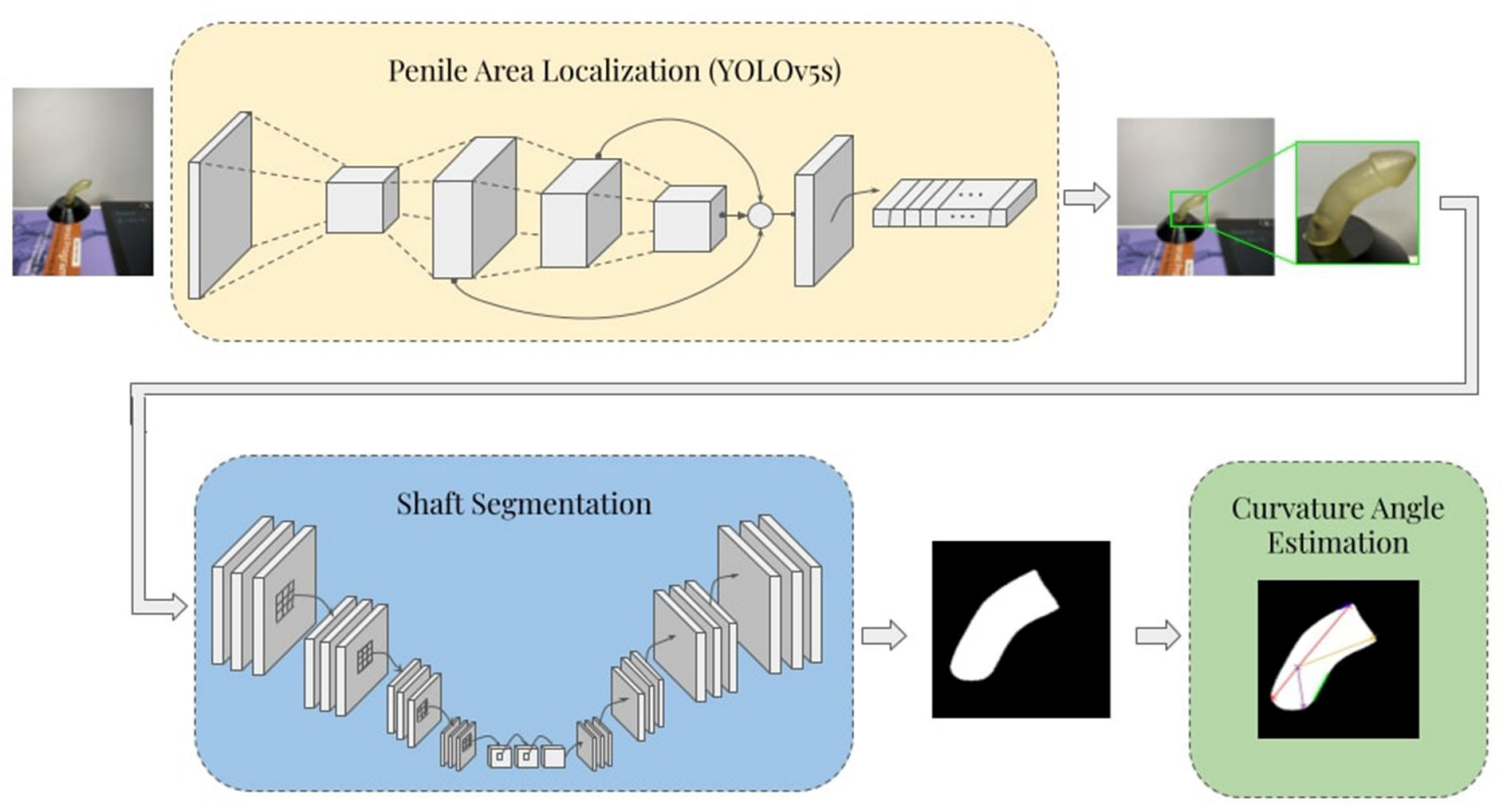
Schematic diagram of the proposed penile curvature angle estimation pipeline.

The proposed framework consists of three stages: automatic penile area localization, shaft segmentation, and curvature angle estimation. In the first stage, the penile area is detected, localized, and extracted from the rest of the image by means of cropping to a predicted bounding box. In the shaft segmentation stage, a deep U-Net based (encoder-decoder) convolutional neural network (CNN) architecture is used to produce binary masks that highlight the penile shaft against the image background. Lastly, the binary mask is post-processed, and curvature angle is determined *via* a custom Hough-Transform-based angle estimation algorithm. The overall schematic diagram of the proposed pipeline is illustrated in Figure 1.

### Dataset description

**Figure 2:**
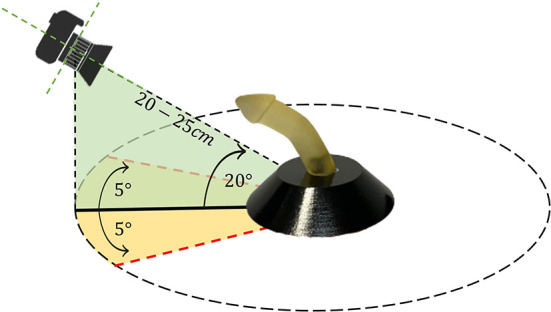
Experimental set-up for model image acquisition.

To develop and validate the proposed framework of PC angle estimation, we used nine realistic 3D-printed penile models with differing uniplanar hinging curvature angles (ranging from 18° to 88°). These models were designed by a 3D model developer and then the associated stereolithography (STL) files were resized to the standard sizes for the pediatric population (1.5 cm wide and 5–6 cm long). Before printing the models, the STL files were smoothed, and sharp edges were shaped into radial curvature that resembled a more typical instance of penile curvature and hypospadias cases in general. Furthermore, these penile phantoms resemble circumcised penis which makes this model easier to be clinically translatable when utilized in circumcised patients as well (Villanueva, 2019). An iPhone 11 Pro Max mobile camera with triple-lens (12 MP resolution) was used to capture images of the penile models. The camera was placed 20–25 cm distant from each model and moved through the horizontal axis (−5°, 5°) and vertical axis (0°, 20°). For each camera position, ~100 images of each model were acquired, resulting in a total dataset of around 900 images. Figure 2 depicts the experimental set-up for collecting images from each model. Horizontal and vertical rotation of the camera with respect to the source was used to evaluate how these variables impacted on PC angle estimation.

### Penile localization

Penile area detection and localization provide a robust basis for estimation of curvature angles. Application of bounding boxes and subsequent cropping of the original images facilitates removal of irrelevant parts to leave only the portions needed for segmentation. This process was performed using the YOLOv5 real-time object detection algorithm (Jocher et al., 2022), which is a new member of the YOLO (You Only Look Once) series which in recent years has been widely applied to the automated assessment of medical images (Redmon et al., 2016; Redmon and Farhadi, 2017, 2018; Bochkovskiy et al., 2020). In YOLOv5, the input image is partitioned into a grid of sub-regions with separate bounding boxes alongside confidence scores and probability labels. The architecture of this network consists of three main parts: backbone, neck, and prediction head. The backbone employs CNN blocks that are known for their strong feature extraction capabilities. Then extracted feature maps are reprocessed to ensure maximum utility in the neck stage. This is typically achieved using multiple upstream and downstream paths along with additional skip connections to ensure high-resolution feature reusability. Next, the head prediction is designed to detect locations and labels of the bounding boxes using the extracted feature maps. Finally, a non-maximum suppression method is used to eliminate overlapping predictions within the same target image (Redmon and Farhadi, 2018). Transfer learning was also used to ensure reliable and efficient training (by initializing weights in the convolutional layers of the backbone with ImageNet pre-trained weights).

### Penile shaft segmentation

Segmentation of the penile shaft was performed using a deep U-Net (encoder-decoder) model. Different state-of-the-art architectures were considered while developing the proposed model, such as U-Net (Ronneberger et al., 2015), U-Net++ (Zhou et al., 2018), and FPN (Lin et al., 2017). Each of these models was investigated with different backbone networks, including ResNet18/50/121/152 (He et al., 2015), DenseNet121/161 (Huang et al., 2016), and inceptionv4 (Szegedy et al., 2016).

The standard U-Net architecture consists of a contracting encoder path and an expanding decoder path, where up-sampling blocks are used instead of pooling operators. In the contracting pathway, the special content of feature maps is gradually diminished while the representational capacity of the context is captured, thus allowing accurate localization through the expanding path and corresponding skipped connections between the encoder and decoder parts. U-net++ introduces modified decoder blocks alongside a redesigned skipped connection scheme, ensuring intensive feature fusion compared to the classical U-net architecture. Feature Pyramid Network (FPN) instead uses a pyramidal hierarchy of feature maps by producing a prediction mask at each spatial resolution. Then, all predicted feature maps are concatenated after imposing a unified spatial resolution through up-sampling. Finally, a 3 × 3 convolutional kernel with a SoftMax activation function is applied to produce the final prediction mask. Binary cross-entropy (BCE) loss function was used in training all the segmentation networks and was defined as:


(1) $$BCE=-\frac{1}{K}\sum_{k}\left[y_{k}\log\left(p\left(x_{k}\right)\right)+\left(1-y_{k}\right)\log\left(1-p\left(x_{k}\right)\right)\right]$$

Here, *yk* is the value of the *kt*h pixel in the binary ground truth segmentation mask, and *p*(*xk*) denotes the SoftMax activation of *kt*h pixel in the predicted segmentation mask.

### Penile curvature angle estimation

**Figure 3:**
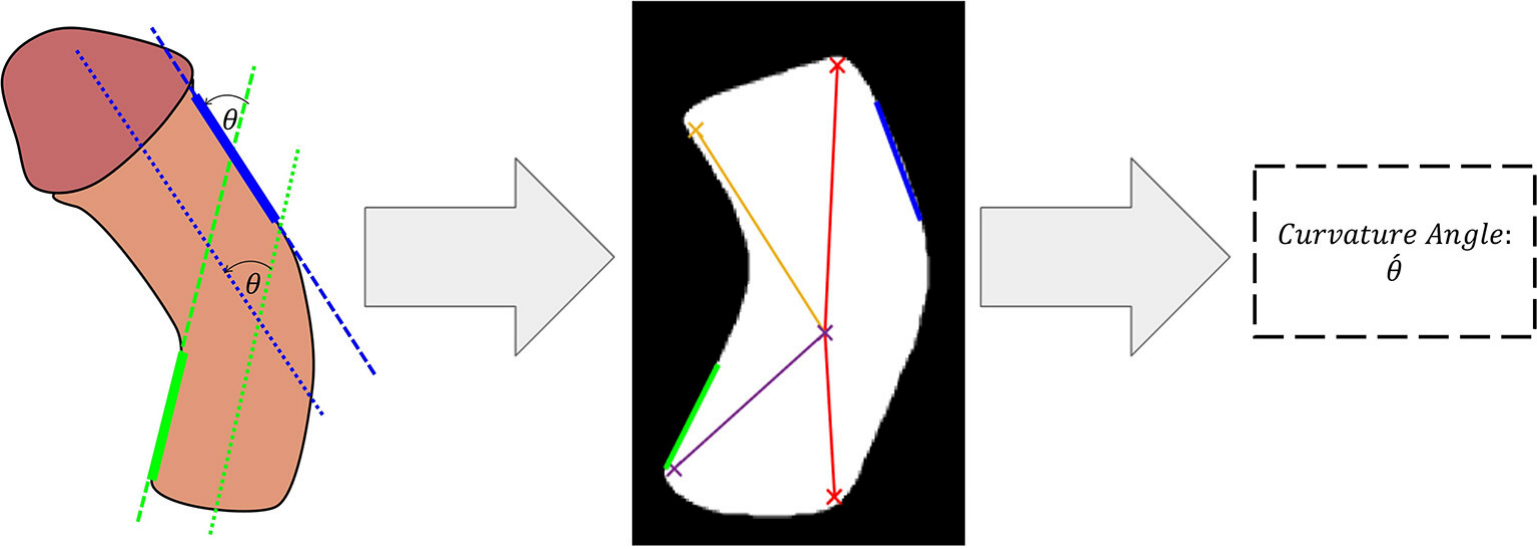
Lines selection method for estimation of curvature angle.

The predicted binary segmentation masks are post-processed by hole filling to compensate for any irregularities in the predicted binary masks. This was achieved by performing a flood-fill operation on the segmented shaft area. The next step applies a multi-stage non-linear median filter with a kernel size of 2 × 2 followed by a 3 × 3 Gaussian smoothing kernel with a variance of 0.25. This post-filtering stage preserved the sharp edges of the image while acting as a smoothing technique for the shaft area. Next, a minimum eigenvalue algorithm was used to detect the four corners of the shaft region. These corners were then used to determine the orientation of the shaft and the curvature angle. In the next step, a Hough-Transform-based line detector was used to fit lines to the inner bottom and the outer upper edges of the shaft. These areas were selected since they consistently aligned with the two main sections of the penile shaft to allow determination of total inclination angle. The detected lines are then filtered and an optimal line from each region is selected as illustrated in Figure 3. Finally, the angle between the two selected lines is estimated using the following equation:


(2) $$\begin{array}{l} \theta =\left[\begin{array}{cc} \arctan|\frac{m_{1}-m_{2}}{1+m_{1}m_{2}}|,\; & \;m_{1},m_{2}\neq \infty \\ \arctan\left(\frac{1}{m_{i}}\right),\; & \begin{array}{c} m_{i}\neq \infty ,\;m_{j}=\infty \\ i,j\epsilon \left\{1,2\right\} \end{array} \end{array}\right] \end{array}$$

Where *m*_1_, *m*_2_ are the slopes of the first and the second line, respectively.

### Experimental setup

The captured images were first center-cropped to restrict the aspect ratio of each image to 1:1. In all experiments, a 5-fold cross-validation scheme was followed, with 20% of the training data used in validating the models and during model selection and hyperparameter tuning. Standard image augmentation techniques were used to increase the number of training examples per fold to 3,500 images. These techniques include translation, rotation, and flipping. It is important to note that the automatically cropped images from the penile area localization step were also used to develop and evaluate the segmentation model. The predicted masks were eventually used to evaluate the curvature angle estimation algorithm. Therefore, the framework is evaluated in two steps: (i) the penile area localization and shaft segmentation and (ii) curvature angle estimation. This strategy was designed to ensure a reliable and unbiased estimation of overall pipeline performance.

For penile area localization, a stochastic gradient descent (SGD) optimizer was used to train the Yolov5s model for 250 epochs with an initial learning rate of 10^−2^ and a momentum of 0.937. Additionally, a batch size of 16, and a weight decay of 5 × 10^−4^ were applied. Adam optimizer was used for development of the shaft segmentation models, with an initial learning rate of 10^−4^ and momentum update of 0.9 and 0.99 for β_1_ and β_2_, respectively. The model was trained for 100 epochs with a mini-batch size of 16 images. An early stopping criterion was employed to avoid overfitting, such that if validation loss does not improve for 15 successive epochs the training will be terminated. An adaptive learning rate scheduler was implemented to reduce the learning rate by a factor of 0.2 after 5 patient epochs where no improvement in validation loss was observed. All experiments conducted in this study were implemented using PyTorch library with Python 3.7 on Intel^®^ Core™ i9-9900K CPU 3.60G Hz and 32.0 GB RAM, with an 8 GB NVIDIA GeForce RTX 2080 SUPER GPU.

### Evaluation metrics

#### Object detection evaluation metric

Mean average precision (mAP) was applied to evaluate performance of the penile area localization network. AP is defined as area under the precision-recall curve, whereas mAP is the mean value of AP over the classes:


(3) $$\begin{array}{l} mAP=\;\frac{1}{n}\;\overset{n}{\underset{i=1}{\sum }}AP_{i}\;for\;n\;classes \end{array}$$

Where the only class in this study is penile area.

mAP provides a solid basis for evaluating object detection models by comparing the ground-truth bounding box to the detected box.

#### Segmentation evaluation metrics

The shaft segmentation networks' performance was mainly examined using three evaluation metrics: Dice Similarity Coefficient (DSC), Intersection over Union (IoU), and model accuracy. These performance metrics are defined as follows:


(4) $$\begin{array}{l} DSC=\frac{2TP}{2TP+FP+FN} \end{array}$$

TP, FP, TN, FN are the counts of true positive, false positive, true negative, and false negative pixels, respectively.


(5) $$\begin{array}{l} IoU=\frac{TP}{TP+FP+FN} \end{array}$$

(6) $$\begin{array}{l} Accuracy=\frac{TP+TN}{TP+FP+TN+FN} \end{array}$$

It should be noted that both IoU and DSC provide a quantitative evaluation of the overlap between predicted and the ground truth segmentation masks, with the primary distinction being that DSC favors true shaft prediction pixels by a factor of 2 compared to IoU.

#### Curvature angle estimation evaluation metrics

Mean absolute error (MAE) was selected as the main assessment scheme for curvature angle estimation. MAE is given by:


(7) $$\begin{array}{l} MAE=\frac{1}{n}\;\overset{n}{\underset{i=1}{\sum }}|{ỹ}_{i}-y_{i}| \end{array}$$

Here *n* is the total number of examples, ỹ_*i*_ is the estimated curvature angle, and *y*_*i*_ is the ground truth curvature angle.

The performance of deep CNNs and the angle estimation algorithm is assessed using different evaluation metrics with a 95% confidence interval (CI) (Mitchell, 1997). Furthermore, the CI (r) for the evaluation metrics is computed as follows:


(8) $$\begin{array}{l} \psi_{95\%\;CI}=1.96\sqrt{\frac{\psi \left(1-\psi \right)}{N}} \end{array}$$

Here ψ is the used metric, and N is the size of the test sample.

## Results

Performance of the proposed AI framework was evaluated quantitatively and qualitatively, including comprehensive assessment for the penile area localization model, shaft segmentation networks, and the curvature angle estimation algorithm.

### Penile area localization

**Figure 4:**
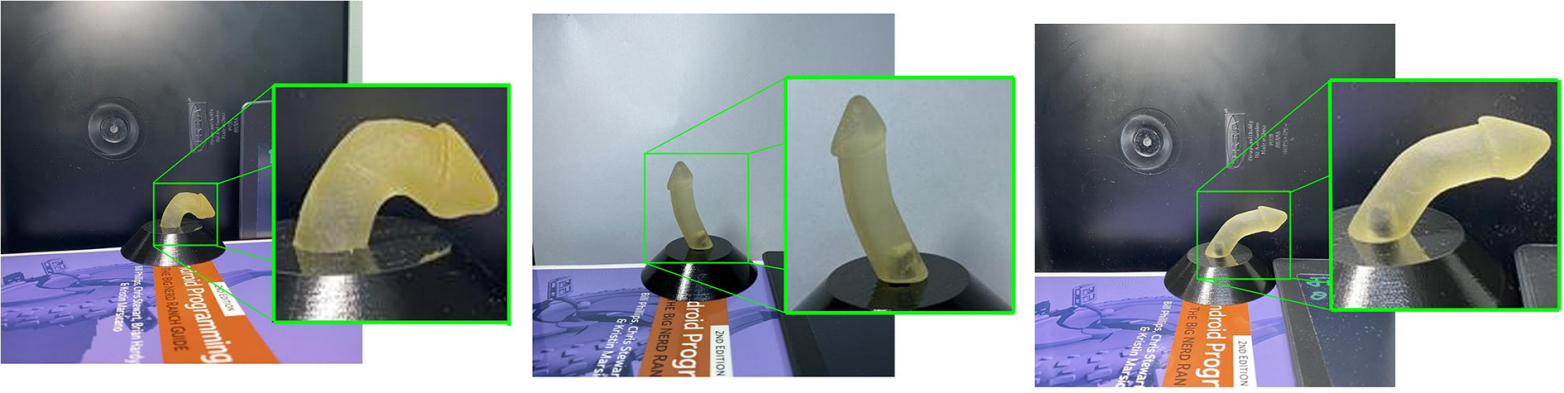
Sample qualitative evaluation of the cropped penile area.

The penile area localization model achieved robust real-time performance in cropping the penile area, with mAP 99.4%. Figure 4 shows a sample qualitative evaluation of model performance.

### Shaft segmentation

**Figure 5:**
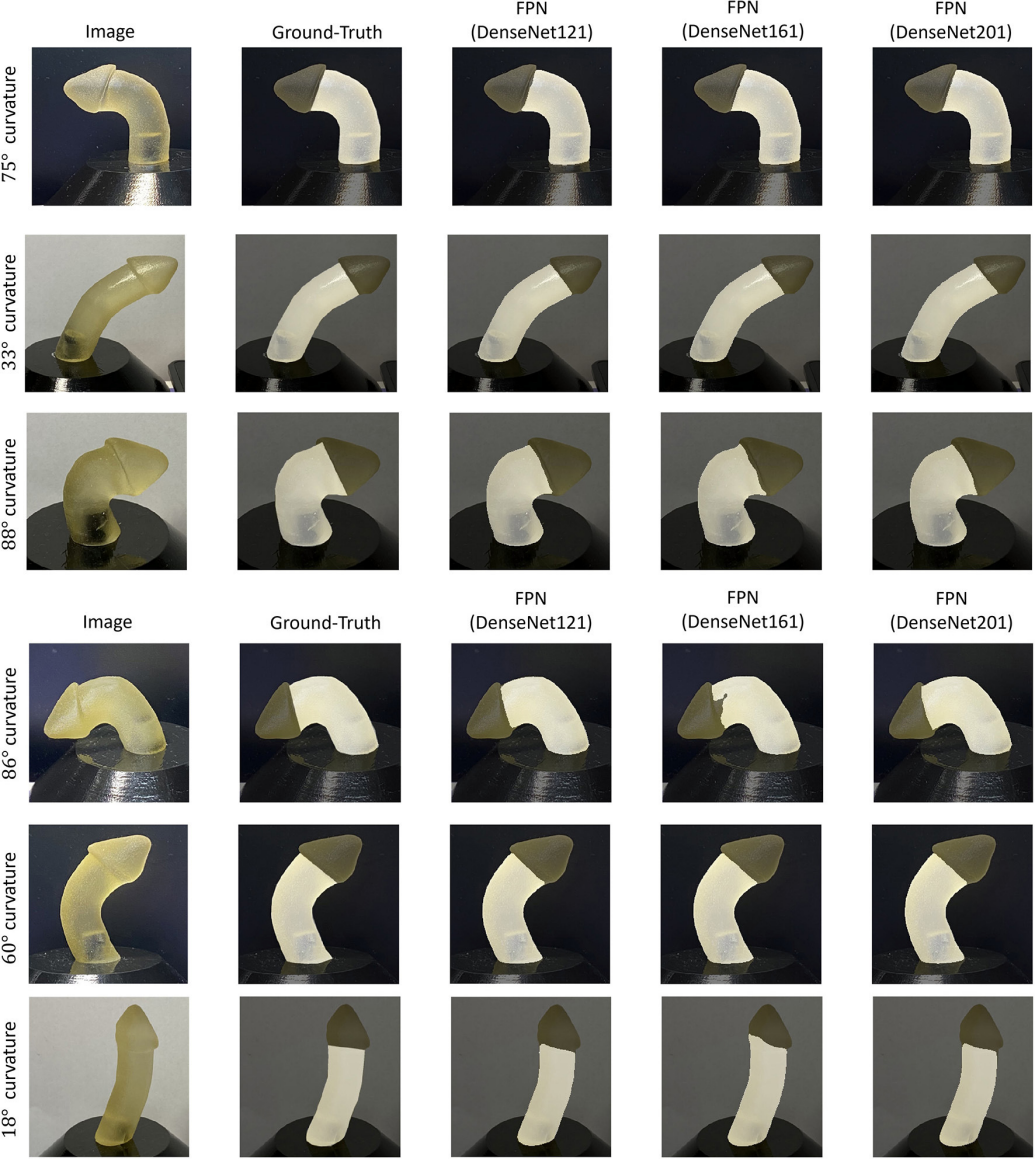
Sample qualitative evaluation of the top-three performing shaft segmentation networks.

Performance of the shaft segmentation models over the test set is illustrated in Table 1. Each model was investigated with seven different backbone architectures varying from shallow to deep. DenseNet encoders achieved superior performance compared with other encoder architectures. This could be due to the extensive connectivity provided by the dense layers and the collective knowledge offered by the preceding layers. This type of encoder block employed together with the FPN model achieved the strongest performance (98.41% DSC and 96.87% IoU). This superior performance may be due to the tendency of FPN models to predict smoother segmentation masks with improved localization performance relative to conventional U-Net and U-Net++ architecture. This tendency could be inherited from the hierarchy architecture of FPN, where low-resolution but semantically strong features are combined with high-resolution feature maps on each of the decoder spatial levels to generate the final prediction mask. In contrast, U-Net and U-Net++ generation of the final prediction mask is solely dependent on the final decoder block of the networks. A qualitative evaluation of the top-three performing networks is presented in Figure 5.

**Table 1:** Performance metrics (%) for penile shaft segmentation, comparing computed over test (unseen) set using three network models and seven encoder architectures.

**Model**	**Encoder**	**Accuracy**	**IoU**	**DSC**
U-Net	ResNet18	98.63 ± 1.91	93.13 ± 8.62	95.90 ± 5.74
	ResNet50	99.29 ± 0.35	96.00 ± 1.78	97.93 ± 0.97
	ResNet152	98.94 ± 0.75	94.39 ± 3.75	97.00 ± 2.11
	DenseNet121	99.40 ± 0.18	96.52 ± 0.96	98.22 ± 0.51
	DenseNet161	99.17 ± 0.48	95.42 ± 2.61	97.59 ± 1.44
	DenseNet201	99.11 ± 0.64	95.34 ± 2.70	97.54 ± 1.51
	InceptionV4	99.01 ± 1.07	95.13 ± 4.10	97.39 ± 2.37
U-Net ++	ResNet18	98.99 ± 1.12	95.14 ± 4.19	97.38 ± 2.47
	ResNet50	98.96 ± 0.88	94.50 ± 4.13	97.04 ± 2.38
	ResNet152	99.06 ± 0.54	94.89 ± 2.98	97.29 ± 1.65
	DenseNet121	99.26 ± 0.40	95.79 ± 2.09	97.81 ± 1.14
	DenseNet161	99.29 ± 0.37	96.06 ± 1.74	97.95 ± 0.95
	DenseNet201	99.15 ± 0.50	95.24 ± 2.64	97.47 ± 1.49
	InceptionV4	99.21 ± 0.59	95.79 ± 2.57	97.80 ± 1.43
FPN	ResNet18	99.38 ± 0.14	96.47 ± 0.67	98.20 ± 0.35
	ResNet50	99.21 ± 0.50	95.74 ± 2.20	97.78 ± 1.22
	ResNet152	98.92 ± 1.13	94.42 ± 4.98	96.95 ± 2.98
	DenseNet121	99.46 ± 0.08	96.87 ± 0.37	98.41 ± 0.20
	DenseNet161	99.43 ± 0.09	96.73 ± 0.56	98.33 ± 0.29
	DenseNet201	99.45 ± 0.11	96.81 ± 0.52	98.37 ± 0.27
	InceptionV4	99.30 ± 0.41	96.10 ± 1.94	97.96 ± 1.10

*Numbers indicate metric value ± standard deviation*.

### Curvature angle estimation

**Figure 6:**
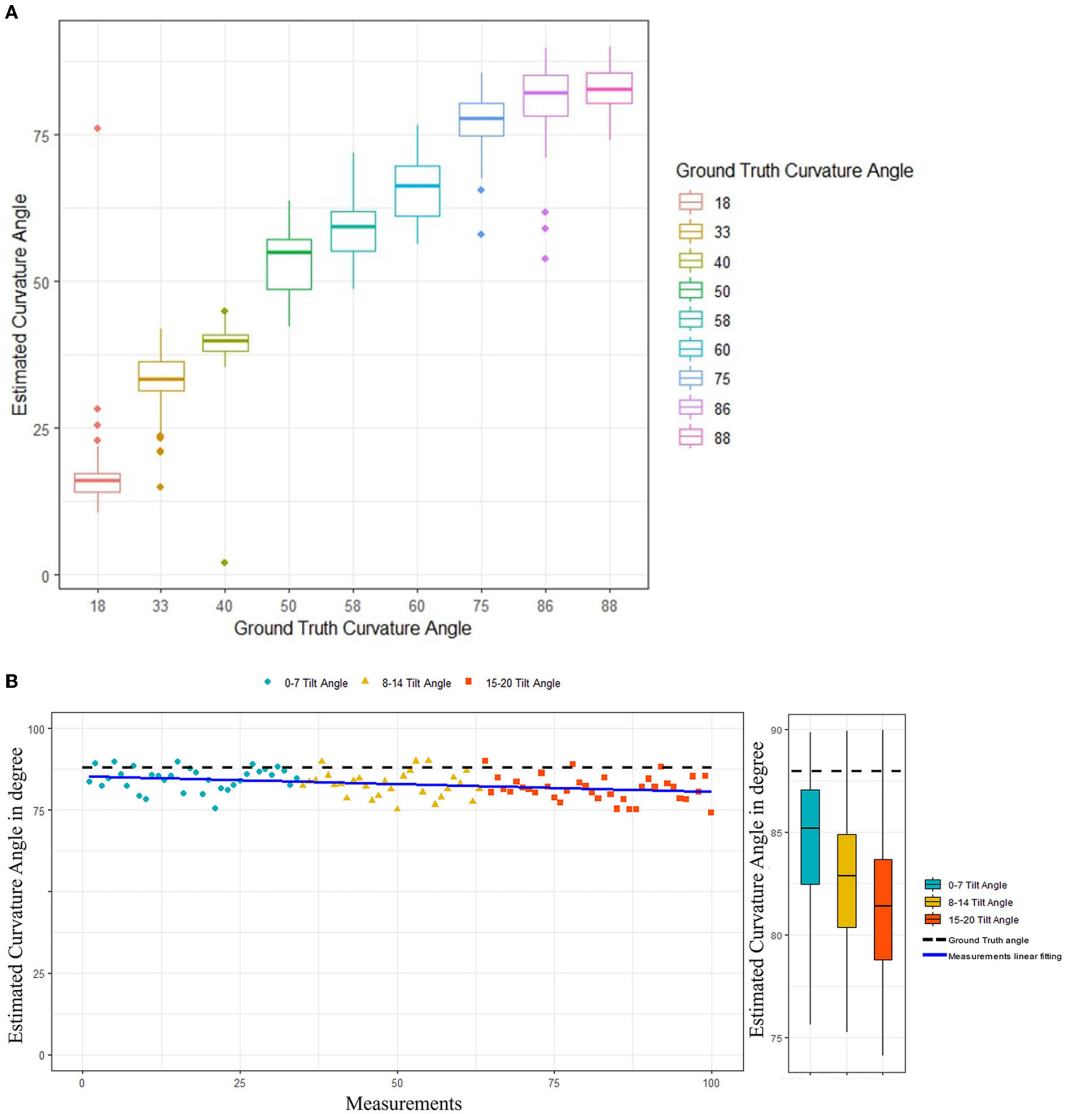
**(A)** Curvature angle estimation algorithms performance. **(B)** Effect of camera tilt angle on performance of the curvature angle estimation algorithm.

Performance of the proposed curvature angle estimation algorithm is presented in Table 2. MAE was considered as the primary performance metric to assess the angle estimation task. An overall error of 8.53° was achieved among different curvature angulations, which illustrates a reliable and accurate angle estimation performance. A maximum of 9.87° MAE was recorded in the case of 86° curvature angulation, mainly due to bias in the mean of measurements when compared to the ground truth value. In contrast, a minimum error of 3.62° was observed in the case of a penile model with 33° curvature angle. Figure 6A illustrates overall performance of the angle estimation technique using a box plot, whereas Figure 6B shows the effect of camera tilt angle on estimation accuracy. The green, yellow and red marks measurements taken with camera angulation <7°, 15°, and 20°, respectively. It is clear that error in angle estimation tends to rise as the camera tilt angle was increased.

**Table 2:** Curvature angle estimation algorithms performance results.

**Measurement (mean ± std)**	**MAE**	**Ground truth**
77.34 ± 4.64	4.22	75
33.18 ± 5.00	3.62	33
82.92 ± 3.91	5.39	88
39.34 ± 4.35	6.15	40
58.72 ± 4.70	3.92	58
53.32 ± 5.49	5.59	50
81.12 ± 6.61	9.87	86
65.78 ± 5.07	6.35	60
15.73 ± 6.09	5.32	18
-	8.53	-

## Discussion

AI provides new opportunities to develop high-fidelity models that allow real-time and precise evaluation of medical images. Machine learning approaches have not previously been used to assess PC severity, therefore we sought to develop and validate an end-to-end AI system that can rapidly emulate the conventional assessments routinely employed by urologists. We designed a fully-automated application that does not require input from the surgeon and instead relies on a novel algorithm that is capable of segmenting the penile shaft to calculate PC angles with high levels of accuracy.

The most widely used intraoperative method for evaluating PC severity is unaided visual inspection (UVI) and approximation of the degree of curvature. While quick and practical to distinguish straight from severely curved penis, intermediate degrees of PC are challenging to quantify objectively. It was recently shown that there is a tendency among hypospadiologists to overestimate or underestimate curvature by an average of 10 degrees on eyeball assessment (Mosa et al., 2022). It should also be noted that recurrent or persistent PC after hypospadias repair is not always obvious, with curvature not being reported by caregivers in 37% (22/60) of cases (and records from the primary surgeons only rarely stating recurrent curvature) (Snodgrass and Bush, 2019). Patients may also overestimate their degree of PC by as much as 20 degrees (Bacal et al., 2009), hence more effective assessment methods are clearly required.

An alternate method for intraoperative evaluation of PC is by goniometer, which requires the device to be placed alongside the erected penis and then fine-tuning its angulation to match PC degree. There are several practical difficulties with employing this method, since it requires maintenance of artificial erection for a prolonged period *via* uninterrupted saline infusion at the same time as compression of surrounding soft tissues, manipulation of the device to match PC angulation, and simultaneous neutralization of penile twist (if any). Accordingly, precision of the goniometer measurements is closely correlated with experience level of the surgeon (Villanueva, 2019).

Mobile applications with the ability to quantify angulated items have been widely used to measure PC in hypospadias patients. These approaches require standardization of numerous variables to achieve satisfactory inter-and intra-observer agreement, but recent reports suggest that smartphone-based measurements were reproducible between observers and correlated well with angle determination by goniometer and protractor (Hsi et al., 2013). Although auto-photography is considered an exciting approach to the 3D assessment of PC, it is not yet clear whether this approach will be limited by significant interobserver discrepancies (Kelâmi, 1983). Objective evaluation of PC correction therefore remains a priority for patient management in this field.

Different camera positions can lead to distortion when converting a 3D object into a 2D image (Ohebshalom et al., 2007), which can be further influenced by the rotational axis and degree of PC. The current study shows that precision of our algorithm was slightly reduced when camera angle deviated from the zero position. The procedure currently followed by the doctors to measure PC when using mobile applications is based on defining three main points: (1) The mid-axis of the distal (upper) limb of the penis, (2) The mid-axis of the proximal (lower) limb of the penis, and the center of the PC where the maximum point of curvature is “thought” to be localized. Our model simulates this process but in an objective manner where the model calculates the degree of the PC based on the estimated degree between the distal and proximal limbs of the penis. Rather than defining the mid-point of curvature as the center of angulation, we instead used a novel algorithm to determine this landmark since some segmentation results were not formed of continuous regions. The method we applied should ensure that defined center points are realistic and match as closely as possible the points manually marked by doctors. The algorithm then defines angulation between both the distal and proximal aspects of PC. Assessment of the mid-axis between ventral and dorsal borders was found to lack accuracy, since these were often irregular in the 3D models and led to errors in angle measurement (albeit similar in extent to those arising during real-life intraoperative assessment due to excess dartos, soft tissues, blood etc.).

This study represents an initial validation performed under controlled conditions, hence it will now be important to assess translatability of our approach to the operating room setting. Nonetheless, this method achieves rapid automatic measurement of PC and could potentially reduce under-anesthesia time for operating teams. The pictures used in our testing dataset were randomly selected from the available image data to span a variety of angulations in addition to evaluate the framework on completely unseen images from a penile model reserved only for testing in each of the performed folds. Since deep learning algorithms rely on high-volume, the next step to increase robustness of this system will be to collect and integrate real-life intraoperative PC images from multiple different medical centers. Since smartphone technology is already being employed by patients and care providers across a range of medical applications, this approach may also present opportunities to further develop patient–provider relationships (Mantica et al., 2020). Employing consistent methods that more accurately define hypospadias phenotype, PC extent, and inform surgical technique should lead to improved outcomes for affected patients. In addition, AI-based methods may yield new data that can guide education, communication, and selection of personalized approaches for hypospadias management.

There are two main etiologies of penile curvature: congenital life birth defects (i.e., hypospadias) and acquired (i.e., Peyronies disease). This model was basically created to optimize the measurement of PC in cases of hypospadias where typically the PC is uniplanar because of ventral corporeal disproportion in such patients. Therefore, this model typically represents realistic deformities encountered in hypospadias cases. On the other hand, some of the acquired PC cases have uniplanar PC where this model can still function and our future optimization of this model will consider multiplanar deformities.

There are however some limitations associated with this study. The compiled dataset only consists of images captured using the 3D printed penile model, which can be considered as a biasing factor since these models cannot span all penile curvature variabilities of the real world, in addition to the absence of real-patient images to validate the proposed framework. Despite these limitations, the main motivation of this study is to present a baseline experiment for an automated penile curvature measurement framework performed under controlled conditions.

## Conclusion

We developed a novel AI-based method for rapid automatic measurement of PC. This method employs deep neural networks to segment the penile shaft prior to assessment and offers accuracy comparable to manual inspection by orthopedic surgeons, but achieved in a far shorter time. Findings from this study suggest that AccuCurve may provide an accurate, reliable, and broadly accessible technique to quantify PC degree, which could overcome numerous shortcomings of current evaluation techniques. Although the framework herein described might not be suitable for clinical use yet, it is our goal that this work will be inspiring to improve and develop automated penile curvature measurement frameworks while presenting a proof-of-concept study that constitute an initially validated experiment performed under controlled conditions. Further improvements to the utility and functionality of this application are likely to happen in near future.

## Data availability statement

The datasets presented in this study can be found in online repositories. The names of the repository/repositories and accession number(s) can be found below: https://github.com/mohamed-ma1707821/AccuCurve, ma1707821.

## Author contributions

TA perceived the concept and wrote the manuscript. MA conducted the AI tasks and helped in writing the manuscript. MC supervised the AI tasks and helped in the analysis and manuscript revision. All authors contributed to the article and approved the submitted version.

## Funding

The authors declare that this study received funding from Hamad Medical Corporation Medical Research Center #20841. The funder was not involved in the study design, collection, analysis, interpretation of data, the writing of this article, or the decision to submit it for publication.

## Conflict of interest

The authors declare that the research was conducted in the absence of any commercial or financial relationships that could be construed as a potential conflict of interest.

## Publisher's note

All claims expressed in this article are solely those of the authors and do not necessarily represent those of their affiliated organizations, or those of the publisher, the editors and the reviewers. Any product that may be evaluated in this article, or claim that may be made by its manufacturer, is not guaranteed or endorsed by the publisher.
